# Trajectories of perioperative serum carcinoembryonic antigen and colorectal cancer outcome: A retrospective, multicenter longitudinal cohort study

**DOI:** 10.1002/ctm2.293

**Published:** 2021-01-21

**Authors:** Zhenhui Li, Chunxia Li, Hongjiang Pu, Xiaolin Pang, Yingyi Wang, Dafu Zhang, Ming Lei, Xianshuo Cheng, Yanrong Zhao, Guiyu Lu, Yingying Ding, Le Cai, Zaiyi Liu, Tao Zhang, Dingyun You

**Affiliations:** ^1^ Department of Radiology Yunnan Cancer Hospital the Third Affiliated Hospital of Kunming Medical University Yunnan Cancer Center Kunming China; ^2^ Department of Colorectal Surgery Yunnan Cancer Hospital Yunnan Cancer Center the Third Affiliated Hospital of Kunming Medical University Kunming China; ^3^ Department of Biostatistics School of Public Health Cheeloo College of Medicine Shandong University Jinan China; ^4^ Department of Oncology Dazhou Central Hospital Dazhou China; ^5^ Department of Radiotherapy the Sixth Affiliated Hospital of Sun Yat‐sen University Guangzhou China; ^6^ Department of Radiology Zhuhai People's Hospital Zhuhai Hospital Affiliated with Jinan University Zhuhai China; ^7^ Department of Clinical Laboratory Medicine Yunnan Cancer Hospital Yunnan Cancer Center the Third Affiliated Hospital of Kunming Medical University Kunming China; ^8^ School of Public Health Kunming Medical University Kunming China; ^9^ Department of Radiology Guangdong Provincial People's Hospital Guangdong Academy of Medical Sciences Guangzhou China

Dear Editor,

Carcinoembryonic antigen (CEA) is regarded as an important tumor marker for colorectal cancer (CRC).^1^, ^2^ The preoperative and postoperative serum CEAs are both associated with the CRC outcome.^3^, ^4^, ^5^ However, the dynamic serum CEA changes after surgery is ignored, and the trajectory of perioperative serum CEA has not been well characterized. The link of it with CRC outcome is unknown.

We used a latent class growth mixed model to distinguish potential CEA dynamic changing trajectories of CRC patients from preoperative to 36 months after surgery using a retrospective, multicenter longitudinal cohort. Then we examined the association of these trajectories with CRC outcome.^6^ A detailed description about the methods can be found in the Supplementary Information.

The number of participants assessed for eligibility and the reasons for exclusion appear in Figure 1A. A total of 2160 patients (1295 [60.0%] male; median [interquartile range, IQR], [49‐65] years) with a median follow‐up time of 43 months (IQR: 32‐60 months) were included. The characteristics of cases are outlined in Table 1. In the analysis, 17 836 individual CEA measurements were investigated. The median number of CEA measurements was 8 (range, 4‐21) (Figure 1B).

**FIGURE 1:**
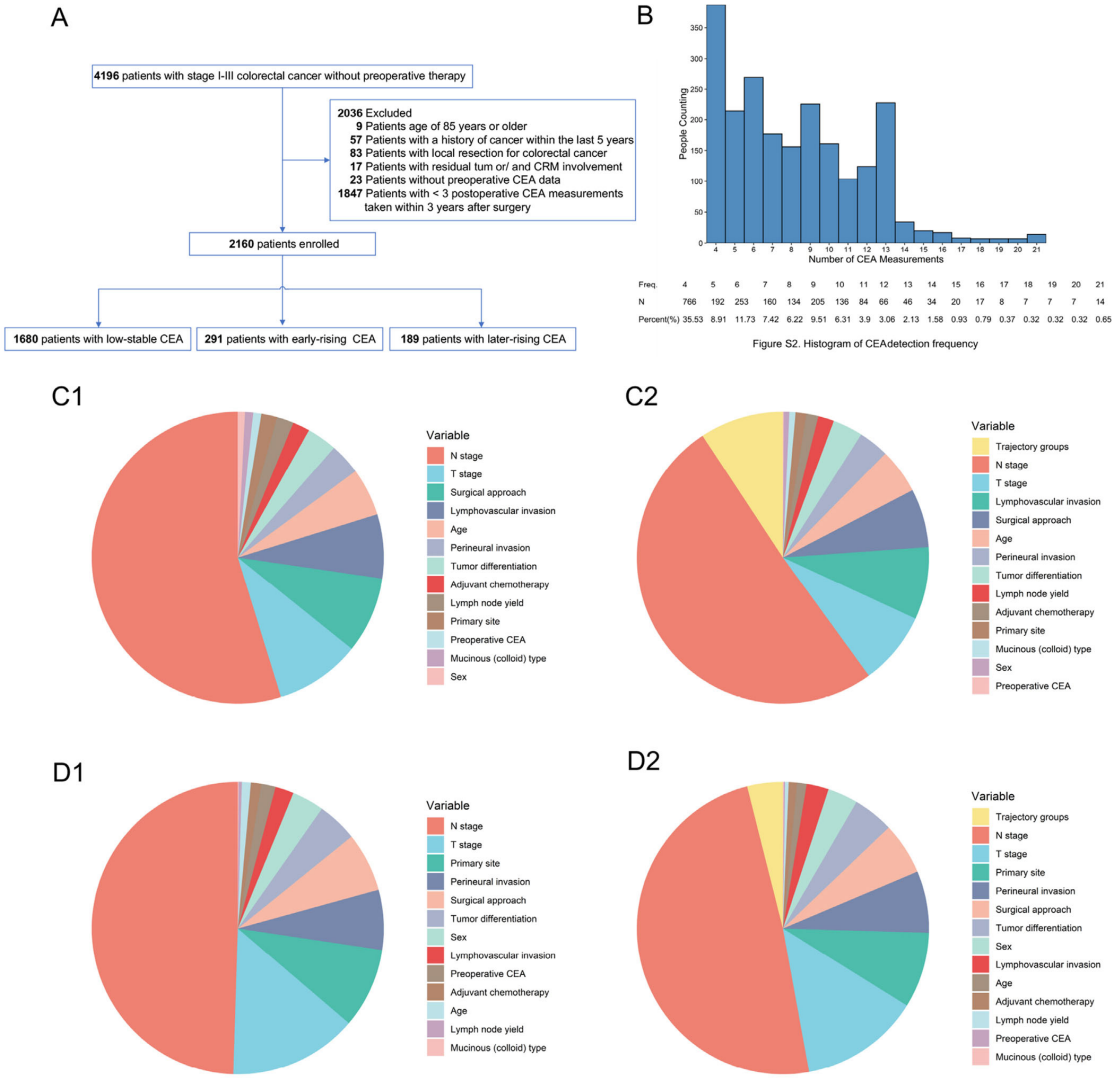
Study flow chart, the histogram of CEA detection frequency, and relative importance of each risk parameter for outcome in colorectal cancer patients. A, study flow chart. This study included 2160 of 4196 patients at three Chinese hospitals. B, Histogram of CEA detection frequency in colorectal cancer patients. C1, Relative importance of each risk parameter for overall survival including clinical parameters. Preoperative CEA: 0.10%; T stage: 9.45%; N stage: 54.79%. C2, relative importance of each risk parameter for overall survival including clinical parameters plus CEA trajectory groups. Preoperative CEA: 0.90%; CEA trajectory groups: 9.28%; T stage: 8.11%; N stage: 50.73%. D1, Relative importance of each risk parameter for recurrence‐free survival including clinical parameters. Preoperative CEA: 1.59%; T stage: 11.24% ; N stage: 49.51%. D2, Relative importance of each risk parameter for recurrence‐free survival including clinical parameters plus CEA trajectory groups. Preoperative CEA: 0.13%; CEA trajectory groups: 3.94%; T stage: 13.26%; N stage: 48.93%. Abbreviations: CEA, carcinoembryonic antigen; HR, hazard ratio;OS, overall survival; RFS, recurrence‐free survival

**TABLE 1:** Characteristics of the cohort at baseline

		Survival status			Trajectory groups			
Variable	Total (n = 2160)	Alive (n = 1918)	Dead (n = 242)	*P* value	Low‐stable group (n = 1680)	Early‐rising group (n = 291)	Later‐rising group (n = 189)	*P* value
**Baseline**								
Age, years^a^	58.0 (49.0, 65.0)	57.0 (49.0, 64.0)	60.0 (50.0, 66.0)	.009	57.0 (49.0, 65.0)	58.0 (49.0, 64.0)	59.0 (50.0, 65.0)	.418
Male, n (%)	1295 (60.0)	1152 (60.1)	143 (59.1)	.825	1039 (61.8)	150 (51.5)	106 (56.1)	
Preoperative CEA, ng/mL^a^	3.8 (2.1, 9.1)	3.6 (2.0, 8.8)	5.8 (2.8, 13.8)	<.001	2.9 (1.8, 5.0)	17.1 (9.1, 33.5)	22.1 (12.9, 41.0)	<.001
Primary site				.051				.236
Colon, n (%)	1220 (56.5)	1098 (57.2)	122 (50.4)		961 (57.2)	151 (51.9)	108 (57.1)	
Rectum, n (%)	940 (43.5)	820 (42.8)	120 (49.6)		719 (42.8)	140 (48.1)	81 (42.9)	
Surgical approach				<.001				.027
Laparoscopic resection, n (%)	1064 (49.3)	981 (51.1)	83 (34.3)		850 (50.6)	137 (47.1)	77 (40.7)	
Open resection, n (%)	1096 (50.7)	937 (48.9)	159 (65.7)		830 (49.4)	154 (52.9)	112 (59.3)	
Tumor differentiation				<.001				.644
Well, n (%)	106 (4.9)	101 (5.3)	5 (2.1)		84 (5.0)	17 (5.8)	5 (2.6)	
Moderate, n (%)	1287 (59.6)	1174 (61.2)	113 (46.7)		1000 (59.5)	173 (59.5)	114 (60.3)	
Poor‐undifferentiated, n (%)	670 (31.0)	563 (29.4)	107 (44.2)		518 (30.8)	92 (31.6)	60 (31.7)	
Unknown, n (%)	97 (4.5)	80 (4.2)	17 (7.0)		78 (4.6)	9 (3.1)	10 (5.3)	
T stage				<.001				<.001
T1, n (%)	90 (4.2)	87 (4.5)	3 (1.2)		80 (4.8)	1 (0.3)	9 (4.8)	
T2, n (%)	275 (12.7)	263 (13.7)	12 (5.0)		240 (14.3)	14 (4.8)	21 (11.1)	
T3, n (%)	1635 (75.7)	1432 (74.7)	203 (83.9)		1246 (74.2)	248 (85.2)	141 (74.6)	
T4, n (%)	160 (7.4)	136 (7.1)	24 (9.9)		114 (6.8)	28 (9.6)	18 (9.5)	
N stage				<.001				<.001
N0, n (%)	1126 (52.1)	1067 (55.6)	59 (24.4)		914 (54.4)	114 (39.2)	98 (51.9)	
N1, n (%)	714 (33.1)	620 (32.3)	94 (38.8)		532 (31.7)	126 (43.3)	56 (29.6)	
N2, n (%)	320 (14.8)	231 (12.0)	89 (36.8)		234 (13.9)	51 (17.5)	35 (18.5)	
AJCC 8th ed. Stage				<.001				<.001
I, n (%)	332 (15.4)	309 (16.1)	23 (9.5)		280 (16.7)	23 (7.9)	29 (15.3)	
II, n (%)	853 (39.5)	803 (41.9)	50 (20.7)		680 (40.5)	100 (34.4)	73 (38.6)	
III, n (%)	975 (45.1)	806 (42.0)	169 (69.8)		720 (42.9)	168 (57.7)	87 (46.0)	
Lymph node yield				.210				.184
<12, n (%)	371 (17.2)	322 (16.8)	49 (20.2)		299 (17.8)	39 (13.4)	33 (17.5)	
≥12, n (%)	1789 (82.8)	1596 (83.2)	193 (79.8)		1381 (82.2)	252 (86.6)	156 (82.5)	
Mucinous (colloid) type, n (%)	140 (6.5)	121 (6.3)	19 (7.9)	.435	106 (6.3)	18 (6.2)	16 (8.5)	.509
Lymphovascular invasion, n (%)	197 (9.1)	148 (7.7)	49 (20.3)	<.001	153 (9.1)	28 (9.6)	16 (8.5)	.911
Perineural invasion, n (%)	120 (5.6)	96 (5.0)	24 (9.9)	.003	95 (5.7)	15 (5.2)	10 (5.3)	.928
Adjuvant chemotherapy, n (%)	1821 (84.3)	1604 (83.6)	217 (89.7)	.019	1403 (83.5)	272 (93.5)	146 (77.2)	<.001
**Follow‐Up**								
Survival time, months^a^	43.2 (32.0, 59.8)	45.6 (34.1, 62.0)	32.7 (22.1, 43.6)	<.001	43.9 (32.3, 60.9)	40.6 (27.6, 57.5)	42.6 (33.1, 56.5)	.045
Recurrence‐free time, months^a^	39.2 (25.3, 56.1)	41.1 (28.7, 58.5)	14.5 (7.1, 28.9)	<.001	39.7 (26.2, 56.8)	37.3 (20.2, 53.7)	37.8 (21.6, 50.9)	.002
Recurrence, n (%)	480 (22.2)	297 (15.5)	183 (75.6)	<.001	337 (20.1)	83 (28.5)	60 (31.7)	<.001

^a^ Data are median (IQR).

We identified three distinct trajectory groups of perioperative CEA, labeled as low‐stable (n = 1680, 77.8%), early‐rising (n = 291, 13.5%), and later‐rising (n = 189, 8.7%) (Figure 2A). In the low‐stable group, the CEA remained within normal range (0‐5.0 ng/mL) from preoperative to 36 months after surgery. In the early‐rising group, CEA declined rapidly from elevated preoperative level (>5.0 ng/mL) toward the normal range within 3 months of surgery, increased rapidly to elevated level (>5.0 ng/mL) (9‐20 months after surgery), and decreased toward the normal range (21‐36 months after surgery). In the later‐rising group, CEA declined rapidly from elevated preoperative level toward the normal range within 3 months of surgery, then kept stable up to 19 months after surgery, and increased slowly to elevated level (19‐35 months after surgery). CEA's trajectories of patients in Yunnan Province and Guangdong Province were similar (Figures 2B and 2C). The three CEA trajectory groups’ characteristics are summarized in Table 1.

**FIGURE 2:**
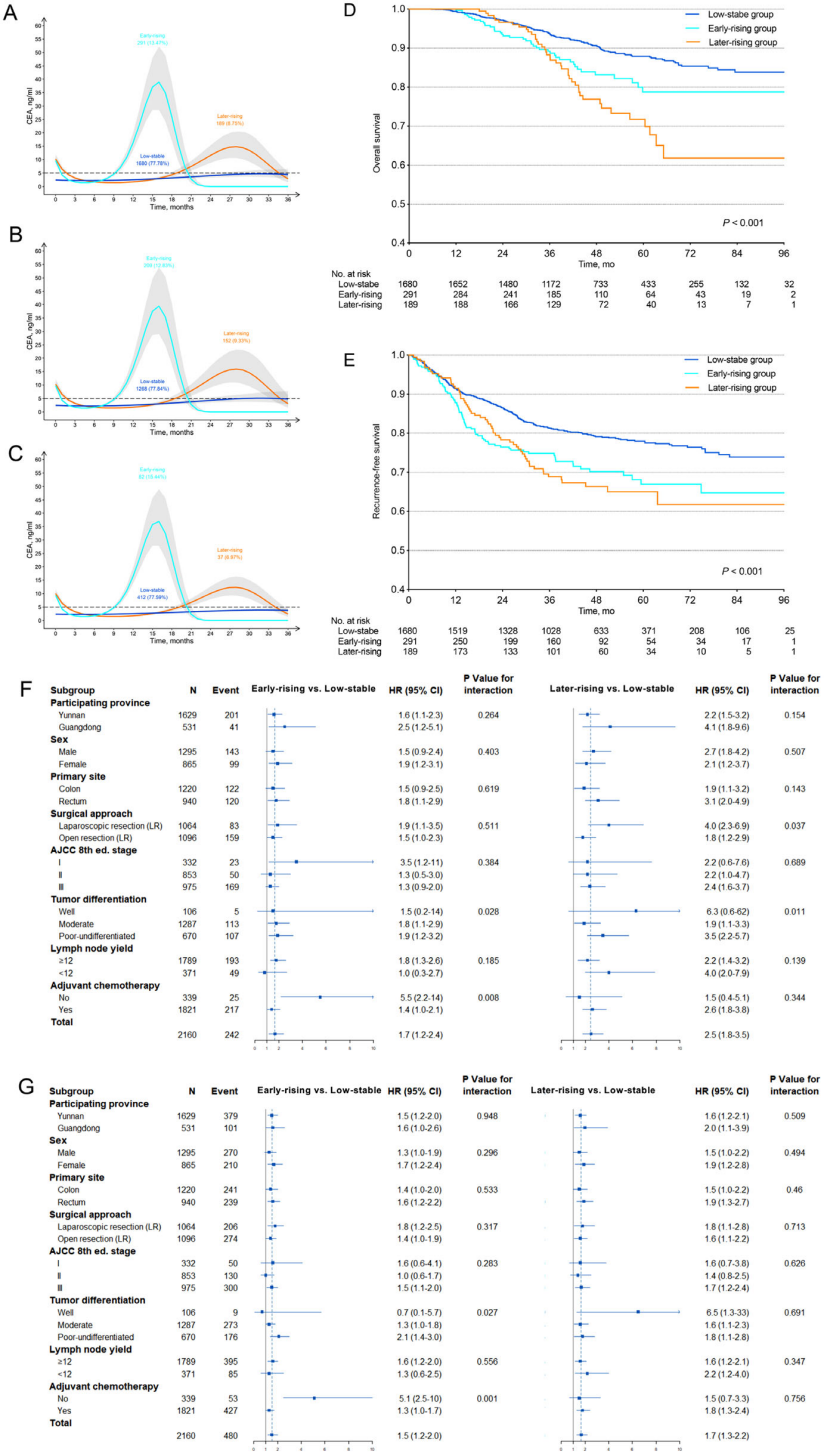
Trajectories of perioperative CEA in colorectal cancer patients, the relation between the trajectories of perioperative CEA and outcome. A, the trajectories in the pooled population. B, The trajectories in population from Yunnan Province. C, The trajectories in population from Guangdong Province. D, Kaplan‐Meier curves for overall survival according to the trajectories of perioperative CEA in colorectal cancer patients. E, Kaplan‐Meier curves for recurrence‐free survival according to the trajectories of perioperative CEA in colorectal cancer patients. F, Forest plot for performance on overall survival of perioperative serum CEA trajectories stratified by clinicopathological features based on the Cox models in colorectal cancer patients. G, Forest plot for performance on overall survival of perioperative serum CEA trajectories stratified by clinicopathological features based on the Cox models in colorectal cancer patients. *P* values for interaction were calculated using the Cox regression model. HR and 95% CIs were given and visually represented by the squares and error bars Abbreviations: CEA, carcinoembryonic antigen; CI, confidence interval; HR: hazard ratio.

We first estimated the over survival (OS) and recurrence‐free survival (RFS) for each trajectory group using the Kaplan‐Meier method. The 5‐year OS rate in the low‐stable group was 87.9% (95% confidence interval [CI]: 85.9%‐89.9%), which was significantly higher than that of the other two groups, as demonstrated in Figure 2D (the early‐rising group: 78.8%, 95% CI: 72.6‐85.4%; the later‐rising group, 71.8%, 95% CI: 64.0%‐80.5%) (*P* < .001). Similar difference of the 5‐year RFS rate among three groups was observed, as shown in Figure 2E (the low‐stable group: 78.0%, 95% CI: 75.8‐80.2%; the early‐rising group: 67.0%, 95% CI: 60.8‐73.8%; the later‐rising group, 65.0%, 95% CI: 57.9‐73.0%) (*P* < .001).

The early‐rising and later‐rising groups both had higher risk of death (hazard ratios [HR]: 1.68, 95% CI: 1.19‐2.36, *P* = .003; HR: 2.46, 95% CI: 1.75‐3.47, *P* < .001, respectively) in unadjusted model, compared with the low‐stable group (Figure 2F). The adjustment resulted in a slight attenuation of the risk estimates both in the demographic‐ and preoperative CEA‐adjusted model and the fully adjusted model (Tables S1 and S2). Similar associations between CEA trajectory groups and RFS were observed (Figure 2G, Tables S1 and S3). Figure S1 showed the example of three patients with different types of CEA trajectories and different prognoses.

To test the risk estimates’ robustness, we used two additional sensitivity analyses. The trajectory group membership still had a positive association with the OS in the frailty model analysis before and after adjustment. And the associations between CEA trajectory groups and RFS before and after adjustment yielded mostly similar results both in the frailty model analysis and the competing risk analysis (Tables S4‐S8).

Finally, to test the robustness of the risk estimates, we performed an exploratory subgroup analysis of OS and RFS according to baseline patients’ characteristics. This subgroup analysis of OS (Figure 2F) and RFS (Figure 2G) found similar results for the overall population.

Our results evidence that, concerning prognosis, the perioperative CEA trajectory rather than preoperative CEA is more instructive. It was an independent prognostic factor in CRC using multivariate analysis. It had an equivalent prognostic value to the classical TNM stage for CRC survival (Figure 1C). In other words, the perioperative CEA trajectory contained more prognostic value than that of the preoperative CEA. It may reflect the biological behavior of CRC at some point (such as preoperative) and the anticancer outcome of tumor treatment, including the surgery and adjuvant chemotherapy.^5^

In this study, we took advantage of the CEA data's availability from multiple follow‐ups of CRC within 3 years after the operation to characterize the perioperative CEA trajectory. It may be a new easy‐to‐use method for exploring the prognostic value of multiple CEA measurements. In clinical applications, doctors only need to observe CEA changes, without calculating CEA change, unlike previous studies.^5^, ^7^, ^8^ It should be noted that not every patient meets all the characteristics of a perioperative CEA trajectory group.^9^ For instance, the early‐rising group also included patients with elevated preoperative CEA, normal CEA within 9 months of surgery, elevated CEA at 10th months after surgery, and unknown CEA levels from then.

Notably, we found that the patients with early‐rising and later‐rising CEA had lower OS and RFS. Hence, our findings may suggest an individualized CEA surveillance strategy. Patients with early‐rising and later‐rising CEA may need more frequent follow‐up testing to detect recurrence at an early stage and increase surgical resection rate with curative intent.^10^ This also needs to be verified by a prospective randomized controlled trial.

In summary, we have identified three distinct trajectories of perioperative CEA, associated with the CRC outcome. This study provides new insights into the prognostic significance of multiple CEA measurements. It emphasizes that patients with the early‐rising or later‐rising CEA may need more frequent follow‐up.

## GUARANTOR OF THE ARTICLE

Dingyun You is the guarantor of the article.

## CONFLICT OF INTEREST

The authors declare that there is no conflict of interest that could be perceived as prejudicing the impartiality of the research reported.

## ETHICS STATEMENT

This multicenter retrospective study was approved by the ethics committee of each participating hospital.

## AUTHOR CONTRIBUTIONS

Concept and design: Dingyun You, Tao Zhang, and Zaiyi Liu. Collection and assembly of data: Zhenhui Li, Chunxia Li, Hongjiang Pu, Xiaolin Pang, Yingyi Wang, Dafu Zhang, Ming Lei, Xianshuo Cheng, Yanrong Zhao, Yingying Ding, and Le Cai. Development of methodology: Dingyun You, Tao Zhang, Zaiyi Liu, Zhenhui Li, Chunxia Li, Hongjiang Pu, Xiaolin Pang, Yingyi Wang, Dafu Zhang, Ming Lei, Xianshuo Cheng, and Yanrong Zhao. Data analysis and interpretation: Dingyun You, Tao Zhang, Zaiyi Liu, Zhenhui Li, Chunxia Li, Hongjiang Pu, Xiaolin Pang, Yingyi Wang, Dafu Zhang, Yingying Ding, and Le Cai. Manuscript writing and final approval of the manuscript: Zhenhui Li, Chunxia Li, Hongjiang Pu, Xiaolin Pang, Yingyi Wang, Dafu Zhang, Ming Lei, Xianshuo Cheng, Yanrong Zhao, Guiyu Lu, Yingying Ding, Le Cai, Zaiyi Liu, Tao Zhang, and Dingyun You.

## FUNDING INFORMATION

Research grants from the National Natural Science Foundation of China (grant numbers: 82073569, 81660545, 81673271, 81960592, 81973147, and 82001986), the Applied Basic Research Projects of Yunnan Province, China (2019FE001‐083, 2018FE001‐065), and Innovative Research Team of Yunnan Province (2019‐6).

## DATA AVAILABILITY STATEMENT

The data underlying this article cannot be shared publicly due to individuals' privacy that participated in the study. The data will be shared on a reasonable request to the corresponding author.

## Supporting information

SUPPORTING INFORMATION

SUPPORTING INFORMATION

SUPPORTING INFORMATION

SUPPORTING INFORMATION

SUPPORTING INFORMATION

SUPPORTING INFORMATION

SUPPORTING INFORMATION

SUPPORTING INFORMATION

SUPPORTING INFORMATION

SUPPORTING INFORMATION

SUPPORTING INFORMATION

SUPPORTING INFORMATION

SUPPORTING INFORMATION

SUPPORTING INFORMATION

SUPPORTING INFORMATION
