# Supplementary figures and images for: Trajectories of perioperative serum carcinoembryonic antigen and colorectal cancer outcome: A retrospective, multicenter longitudinal cohort study
Source: Clin Transl Med. 2021 Jan 21;11(2):e293. doi: 10.1002/ctm2.293 (PMC7818970; doi:10.1002/ctm2.293)

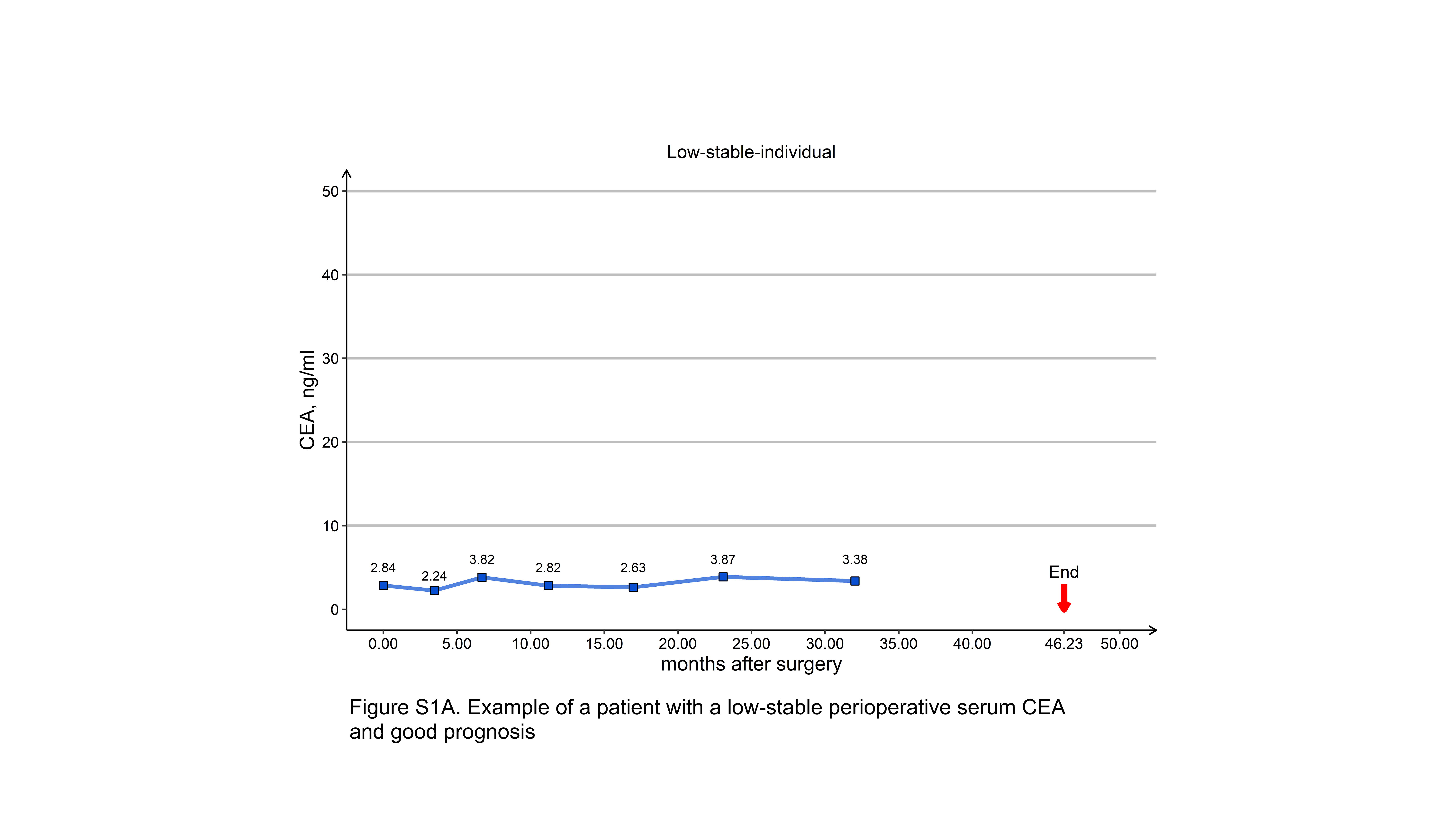

Supplement: Supplementary file 1 — SUPPORTING INFORMATION [file CTM2-11-e293-s001.tiff]

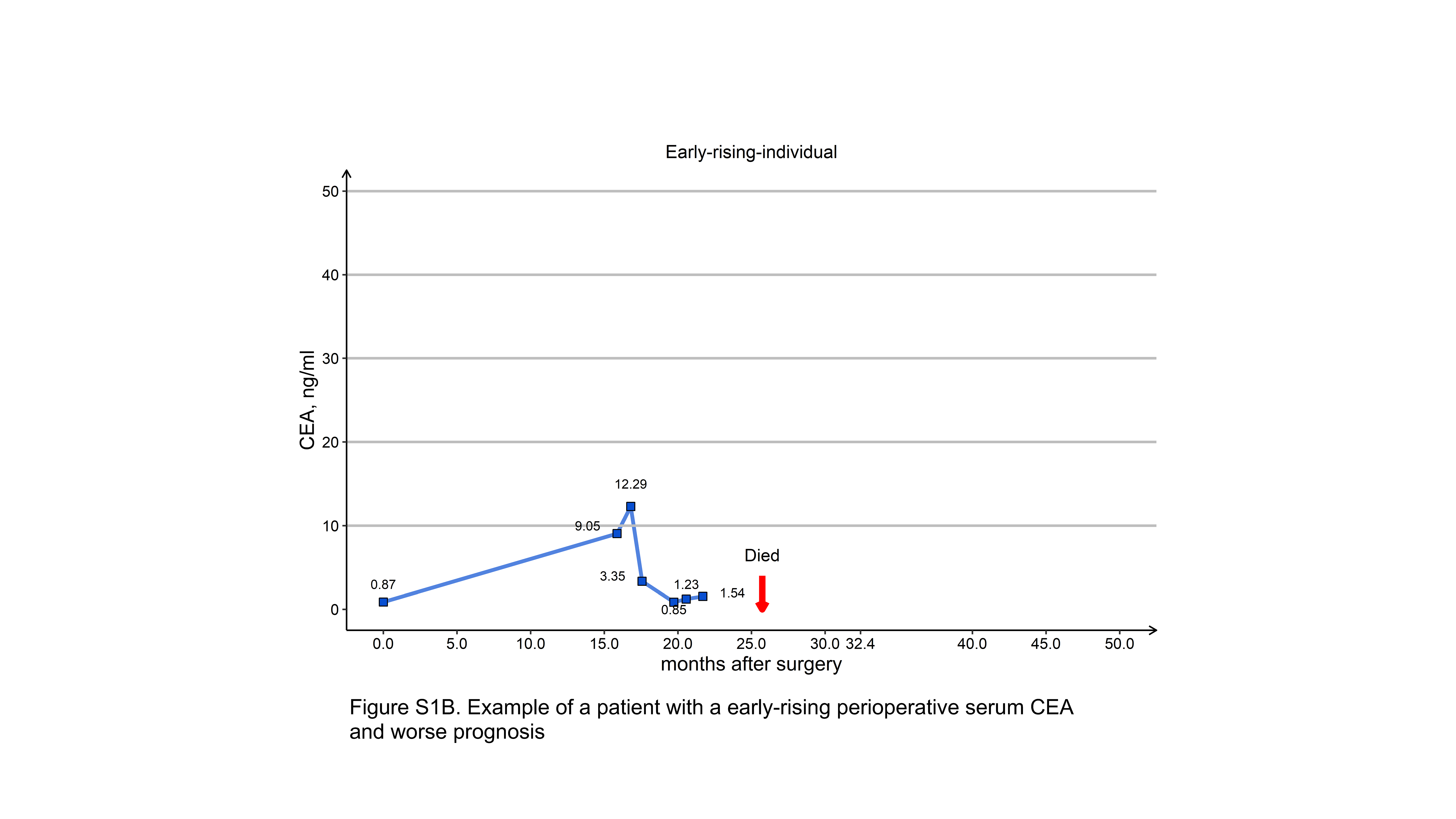

Supplement: Supplementary file 2 — SUPPORTING INFORMATION [file CTM2-11-e293-s002.tiff]

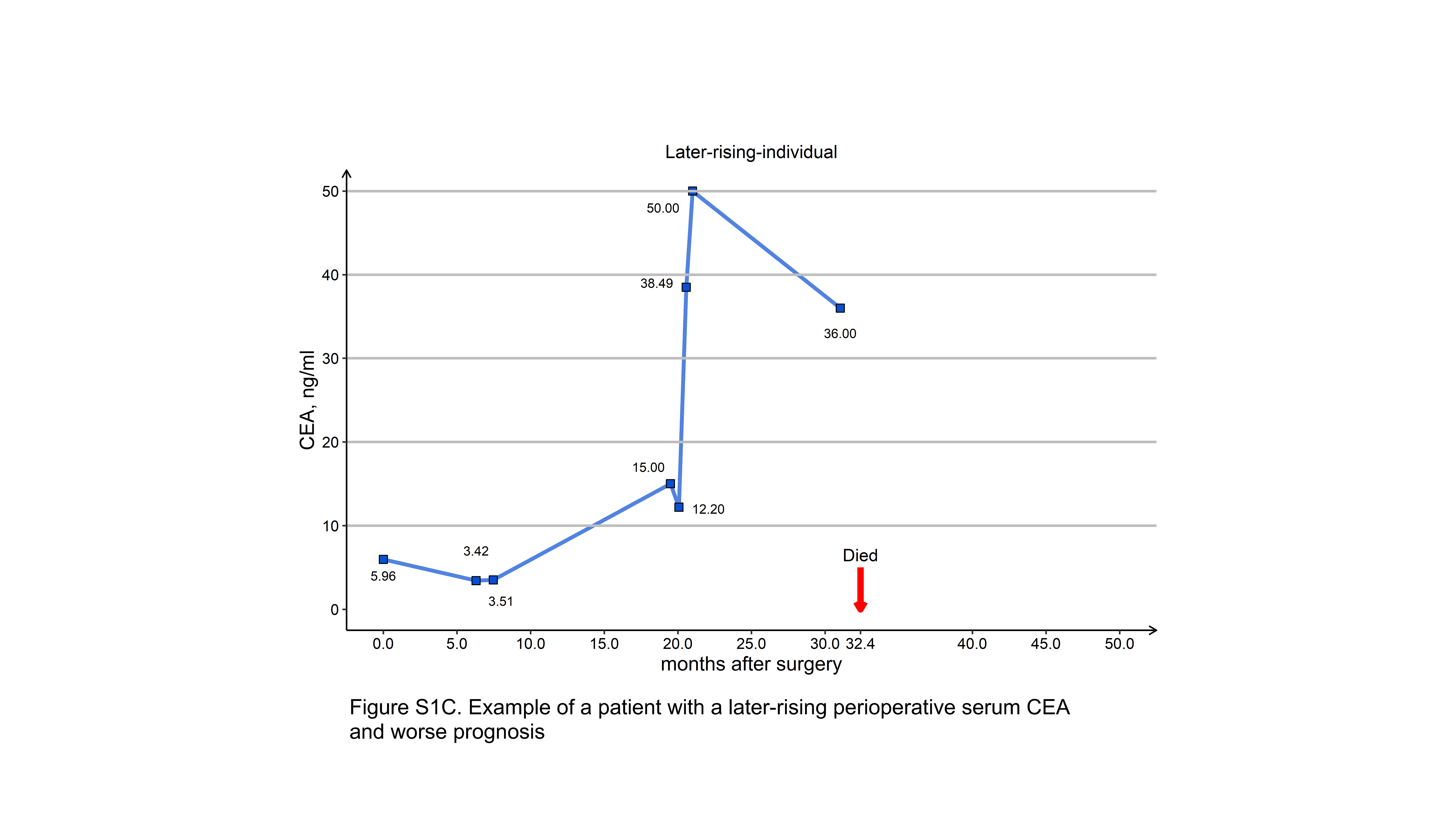

Supplement: Supplementary file 3 — SUPPORTING INFORMATION [file CTM2-11-e293-s003.tiff]
