# Supplementary material for: Trajectories of perioperative serum carcinoembryonic antigen and colorectal cancer outcome: A retrospective, multicenter longitudinal cohort study
Source: Clin Transl Med. 2021 Jan 21;11(2):e293. doi: 10.1002/ctm2.293 (PMC7818970; doi:10.1002/ctm2.293)
Supplement: Supplementary file 6 — SUPPORTING INFORMATION [file CTM2-11-e293-s006.docx]

**Table S2. Trajectories of serum CEA and overall survival (A Cox model)**

|  | Model1 | Model2 | Model3 |
| --- | --- | --- | --- |
| Trajectory groups |  |  |  |
| Low-stable | Reference | Reference | Reference |
| Early-rising | 1.68 (1.19-2.36) | 1.64 (1.15-2.33) | 1.45 (1.02-2.08) |
| Later-rising | 2.46 (1.75-3.47) | 2.36 (1.63-3.43) | 2.33 (1.59-3.43) |
| Covariates |  |  |  |
| Age, years |  | 1.01 (1.00-1.02) | 1.02 (1.01-1.03) |
| Preoperative CEA, ng/ml |  | 1.00 (1.00-1.00) | 1.00 (1.00-1.00) |
| Sex |  |  |  |
| Male |  | Reference | Reference |
| Female |  | 1.03 (0.80-1.34) | 1.10 (0.84-1.43) |
| Primary site |  |  |  |
| Colon |  |  | Reference |
| Rectum |  |  | 1.13 (0.87-1.47) |
| Surgical approach |  |  |  |
| Laparoscopic resection |  |  | Reference |
| Open resection |  |  | 1.52 (1.15-2.01) |
| Tumor differentiation |  |  |  |
| Well |  |  | Reference |
| Moderate |  |  | 1.86 (0.68-5.11) |
| Poor-undifferentiated |  |  | 2.66 (0.96-7.33) |
| AJCC 8th ed. Stage |  |  |  |
| I |  |  | Reference |
| II |  |  | 0.81 (0.47-1.38) |
| III |  |  | 2.47 (1.51-4.06) |
| Lymph node yield |  |  |  |
| ≥12 |  |  | Reference |
| <12 |  |  | 1.16(0.84-1.61) |
| Mucinous (colloid) type |  |  |  |
| No |  |  | Reference |
| Yes |  |  | 1.46 (0.89-2.41) |
| Lymphovascular invasion |  |  |  |
| No |  |  | Reference |
| Yes |  |  | 2.31 (1.63-3.29) |
| Perineural invasion |  |  |  |
| No |  |  | Reference |
| Yes |  |  | 1.93 (1.21-3.07) |
| Adjuvant chemotherapy |  |  |  |
| No |  |  | Reference |
| Yes |  |  | 1.01 (0.63-1.63) |

Note: Model 1 was an unadjusted model. Model 2 was a demographic- and preoperative CEA- adjusted model. Model 3 was a fully adjusted model.
