# Supplementary material for: Trajectories of perioperative serum carcinoembryonic antigen and colorectal cancer outcome: A retrospective, multicenter longitudinal cohort study
Source: Clin Transl Med. 2021 Jan 21;11(2):e293. doi: 10.1002/ctm2.293 (PMC7818970; doi:10.1002/ctm2.293)
Supplement: Supplementary file 8 — SUPPORTING INFORMATION [file CTM2-11-e293-s008.docx]

**Table S4. Trajectories of serum CEA and risk of outcomes (A frailty model analysis)**

| Outcome | | Model 1 | |  | Model 2 | |  | Model 3 | |
| --- | --- | --- | --- | --- | --- | --- | --- | --- | --- |
|  |  | Hazard Ratio (95% CI) | *P* Value |  | Hazard Ratio (95% CI) | *P* Value |  | Hazard Ratio (95% CI) | *P* Value |
| Death | |  |  |  |  |  |  |  |  |
|  | Early-rising vs. Low-stable | 1.69 (1.20-2.38) | 0.002 |  | 1.65 (1.16-2.34) | 0.006 |  | 1.45 (1.02-2.08) | 0.040 |
|  | Later-rising vs. Low-stable | 2.42 (1.71-3.42) | < 0.001 |  | 2.31 (1.59-3.36) | < 0.001 |  | 2.33 (1.59-3.43) | < 0.001 |
| Recurrence | |  |  |  |  |  |  |  |  |
|  | Early-rising vs. Low-stable | 1.55 (1.22-1.97) | < 0.001 |  | 1.47 (1.14-1.88) | 0.003 |  | 1.30 (1.01-1.66) | 0.042 |
|  | Later-rising vs. Low-stable | 1.66 (1.26-2.18) | < 0.001 |  | 1.54 (1.14-2.07) | 0.005 |  | 1.52 (1.12-2.05) | 0.006 |

Note:

CEA: carcinoembryonic antigen; CI: confidence interval.

Model 1 was unadjusted.

Model 2 was adjusted for age, sex (female vs. male) and preoperative CEA.

Model 3 was adjusted for age, sex (female vs. male) and preoperative CEA, primary site (rectum vs. colon), surgical approach (open resection vs. laparoscopic resection), tumor differentiation (poor-undifferentiated & moderate vs. well), pathology stage (III→ I), lymph node yield (≥12 vs. <12) mucinous (colloid) type (yes vs. no), lymphovascular invasion (yes vs. no), perineural invasion (yes vs. no) , and adjuvant chemotherapy (yes vs. no).
