# Supplementary material for: Trajectories of perioperative serum carcinoembryonic antigen and colorectal cancer outcome: A retrospective, multicenter longitudinal cohort study
Source: Clin Transl Med. 2021 Jan 21;11(2):e293. doi: 10.1002/ctm2.293 (PMC7818970; doi:10.1002/ctm2.293)
Supplement: Supplementary file 9 — SUPPORTING INFORMATION [file CTM2-11-e293-s009.docx]

**Table S5. Trajectories of serum CEA and risk of outcomes (A competitive risk model)**

| Outcome | | Model 1 | |  | Model 2 | |  | Model 3 | |
| --- | --- | --- | --- | --- | --- | --- | --- | --- | --- |
|  |  | Hazard Ratio (95% CI) | *P* Value |  | Hazard Ratio (95% CI) | *P* Value |  | Hazard Ratio (95% CI) | *P* Value |
| Recurrence | |  |  |  |  |  |  |  |  |
|  | Early-rising vs. Low-stable | 1.66 (1.27-2.17) | < 0.001 |  | 1.55 (1.15-2.09) | 0.004 |  | 1.50 (1.11-2.02) | 0.010 |
|  | Later-rising vs. Low-stable | 1.53 (1.20-1.95) | 0.001 |  | 1.45 (1.12-1.88) | 0.005 |  | 1.28 (1.00-1.66) | 0.053 |

Note:

CEA: carcinoembryonic antigen; CI: confidence interval.

Model 1 was unadjusted.

Model 2 was adjusted for age, sex (female vs. male) and preoperative CEA.

Model 3 was adjusted for age, sex (female vs. male) and preoperative CEA, primary site (rectum vs. colon), surgical approach (open resection vs. laparoscopic resection), tumor differentiation (poor-undifferentiated & moderate vs. well), pathology stage (III→ I), lymph node yield (≥12 vs. <12) mucinous (colloid) type (yes vs. no), lymphovascular invasion (yes vs. no), perineural invasion (yes vs. no) , and adjuvant chemotherapy (yes vs. no).
