# Supplementary material for: Trajectories of perioperative serum carcinoembryonic antigen and colorectal cancer outcome: A retrospective, multicenter longitudinal cohort study
Source: Clin Transl Med. 2021 Jan 21;11(2):e293. doi: 10.1002/ctm2.293 (PMC7818970; doi:10.1002/ctm2.293)
Supplement: Supplementary file 11 — SUPPORTING INFORMATION [file CTM2-11-e293-s011.docx]

**Table S7. Trajectories of serum CEA and recurrence-free survival (A frailty model analysis)**

|  | Model1 | Model2 | Model3 |
| --- | --- | --- | --- |
| Trajectory groups |  |  |  |
| Low-stable | Reference | Reference | Reference |
| Early-rising | 1.55 (1.22-1.97) | 1.47 (1.14-1.88) | 1.30 (1.01-1.66) |
| Later-rising | 1.66 (1.26-2.18) | 1.54 (1.14-2.07) | 1.52 (1.12-2.05) |
| Covariates |  |  |  |
| Age, years |  | 1.00 (1.00-1.01) | 1.01 (1.00-1.01) |
| Preoperative CEA, ng/ml |  | 1.00 (1.00-1.00) | 1.00 (1.00-1.00) |
| Sex |  |  |  |
| Male |  | Reference | Reference |
| Female |  | 1.16 (1.00-1.39) | 1.20 (1.00-1.45) |
| Primary site |  |  |  |
| Colon |  |  | Reference |
| Rectum |  |  | 1.37 (1.14-1.66) |
| Surgical approach |  |  |  |
| Laparoscopic resection |  |  | Reference |
| Open resection |  |  | 1.30 (1.08-1.57) |
| Tumor differentiation |  |  |  |
| Well |  |  | Reference |
| Moderate |  |  | 2.44 (1.25-4.78) |
| Poor-undifferentiated |  |  | 2.71 (1.38-5.35) |
| AJCC 8th ed. Stage |  |  |  |
| I |  |  | Reference |
| II |  |  | 1.01 (0.71-1.44) |
| III |  |  | 2.14 (1.52-3.01) |
| Lymph node yield |  |  |  |
| ≥12 |  |  | Reference |
| <12 |  |  | 0.99(0.78-1.27) |
| Mucinous (colloid) type |  |  |  |
| No |  |  | Reference |
| Yes |  |  | 1.00 (0.67-1.47) |
| Lymphovascular invasion |  |  |  |
| No |  |  | Reference |
| Yes |  |  | 1.52 (1.16-2.00) |
| Perineural invasion |  |  |  |
| No |  |  | Reference |
| Yes |  |  | 1.92 (1.38-2.66) |
| Adjuvant chemotherapy |  |  |  |
| No |  |  | Reference |
| Yes |  |  | 1.12 (0.81-1.56) |

Note: Model 1 was an unadjusted model. Model 2 was a demographic- and preoperative CEA- adjusted model. Model 3 was a fully adjusted model.
