# Supplementary material for: Trajectories of perioperative serum carcinoembryonic antigen and colorectal cancer outcome: A retrospective, multicenter longitudinal cohort study
Source: Clin Transl Med. 2021 Jan 21;11(2):e293. doi: 10.1002/ctm2.293 (PMC7818970; doi:10.1002/ctm2.293)
Supplement: Supplementary file 12 — SUPPORTING INFORMATION [file CTM2-11-e293-s012.docx]

**Table S8. Trajectories of serum CEA and recurrence-free survival (A competitive risk model)**

|  | Model1 | Model2 | Model3 |
| --- | --- | --- | --- |
| Trajectory groups |  |  |  |
| Low-stable | Reference | Reference | Reference |
| Early-rising | 1.53 (1.20-1.95) | 1.47 (1.14-1.88) | 1.28 (1.00-1.66) |
| Later-rising | 1.66 (1.27-2.17) | 1.54 (1.14-2.07) | 1.50 (1.11-2.02) |
| Covariates |  |  |  |
| Age, years |  | 1.00 (1.00-1.01) | 1.01 (1.00-1.01) |
| Preoperative CEA, ng/ml |  | 1.00 (1.00-1.00) | 1.00 (1.00-1.00) |
| Sex |  |  |  |
| Male |  | Reference | Reference |
| Female |  | 1.16 (0.97-1.40) | 1.21 (1.00-1.47) |
| Primary site |  |  |  |
| Colon |  |  | Reference |
| Rectum |  |  | 1.37 (1.13-1.65) |
| Surgical approach |  |  |  |
| Laparoscopic resection |  |  | Reference |
| Open resection |  |  | 1.30 (1.07-1.56) |
| Tumor differentiation |  |  |  |
| Well |  |  | Reference |
| Moderate |  |  | 2.44 (1.23-4.84) |
| Poor-undifferentiated |  |  | 2.71 (1.36-5.41) |
| AJCC 8th ed. Stage |  |  |  |
| I |  |  | Reference |
| II |  |  | 1.02 (0.70-1.49) |
| III |  |  | 2.14 (1.48-3.10) |
| Lymph node yield |  |  |  |
| ≥12 |  |  | Reference |
| <12 |  |  | 0.99(0.78-1.26) |
| Mucinous (colloid) type |  |  |  |
| No |  |  | Reference |
| Yes |  |  | 1.00 (0.67-1.47) |
| Lymphovascular invasion |  |  |  |
| No |  |  | Reference |
| Yes |  |  | 0.98 (0.65-1.47) |
| Perineural invasion |  |  |  |
| No |  |  | Reference |
| Yes |  |  | 1.89 (1.34-2.65) |
| Adjuvant chemotherapy |  |  |  |
| No |  |  | Reference |
| Yes |  |  | 1.12 (0.79-1.59) |

Note: Model 1 was an unadjusted model. Model 2 was a demographic- and preoperative CEA- adjusted model. Model 3 was a fully adjusted model.
