# Supplementary material for: Trajectories of perioperative serum carcinoembryonic antigen and colorectal cancer outcome: A retrospective, multicenter longitudinal cohort study
Source: Clin Transl Med. 2021 Jan 21;11(2):e293. doi: 10.1002/ctm2.293 (PMC7818970; doi:10.1002/ctm2.293)
Supplement: Supplementary file 13 — SUPPORTING INFORMATION [file CTM2-11-e293-s013.docx]

**Table S9. Latent class growth mixture models result of model fitting process**

| No. Latent class | Polynomial degree | Log-Lik | BIC | Participants per class (%) | Mean posterior probabilities | Posterior probabilities > 0.7 (%) |
| --- | --- | --- | --- | --- | --- | --- |
| 1 | Linear | -17667 | 35388 | 100 | NA | NA |
| 1 | Quadratic | -17431 | 34939 | 100 | NA | NA |
| 1 | Cubic | -17132 | 34365 | 100 | NA | NA |
| 2 | Linear | -17484 | 35044 | 2.78/ 97.22 | 0.87/ 0.99 | 83.33/ 99.14 |
| 2 | Quadratic | -16725 | 33557 | 11.71/ 88.29 | 0.88/ 0.98 | 82.61/ 98.11 |
| 2 | Cubic | -16039 | 32217 | 15.97/ 84.03 | 0.90/ 0.98 | 87.54/ 97.52 |
| 3 | Linear | -17396 | 34892 | 3.06/ 94.72/ 2.22 | 0.85/ 0.99/ 0.87 | 75.76/ 98.39/ 75 |
| 3 | Quadratic | -16500 | 33138 | 9.54/ 7.69/ 82.78 | 0.84/ 0.79/ 0.95 | 78.64/ 63.86/ 95.08 |
| **3** | **Cubic** | **-15616** | **31409** | **8.75/ 77.78/ 13.47** | **0.84/ 0.97/ 0.87** | **74.07/ 96.19/ 85.91** |
| 4 | Linear | -17217 | 34557 | 3.56/ 1.53/ 88.75/ 6.16 | 0.85/ 0.88/ 0.96/ 0.88 | 80.52/ 84.85/ 97.24/ 82.71 |
| 4 | Quadratic | -16315 | 32800 | 8.43/ 7.78/ 80.05/ 3.75 | 0.86/ 0.79/ 0.94/ 0.90 | 79.67/ 66.67/ 94.62/ 82.72 |
| 4 | Cubic | -15411 | 31038 | 4.49/ 10.00/ 12.41/ 73.1 | 0.87/ 0.84/ 0.77/ 0.95 | 82.47/ 77.78/ 64.18/ 94.11 |
| 5 | Linear | -17242 | 34629 | 2.36/ 3.56/ 86.48/ 7.59/ 0 | 0.83/ 0.72/ 0.83/ 0.88/ NA | 72.55/ 49.35/ 93.36/ 82.32/ NA |
| 5 | Quadratic | -16199 | 32598 | 9.31/ 3.56/ 1.67/ 78.47/ 6.99 | 0.80/ 0.92/ 0.84/ 0.93/ 0.75 | 69.65/ 84.42/ 69.44/ 93.27/ 58.94 |
| 5 | Cubic | -15362 | 30978 | 4.54/ 5.23/ 19.72/ 58.15/ 12.36 | 0.86/ 0.84/ 0.66/ 0.79/ 0.77 | 81.63/ 76.99/ 36.15/ 71.26/ 66.67 |

Note:

No. Latent class: latent class number of the model;

Log-Lik: the maximum Log-Likelihood;

BIC: the Bayesian information Criterion;

% Participants per class: proportion of participants per class;

The best fitting model is highlighted in bold characters. (NA: not applicable).
