# Supplementary material for: Trajectories of perioperative serum carcinoembryonic antigen and colorectal cancer outcome: A retrospective, multicenter longitudinal cohort study
Source: Clin Transl Med. 2021 Jan 21;11(2):e293. doi: 10.1002/ctm2.293 (PMC7818970; doi:10.1002/ctm2.293)
Supplement: Supplementary file 14 — SUPPORTING INFORMATION [file CTM2-11-e293-s014.docx]

**Table S10. Parameter estimates for the best fitting 3-class cubic latent class growth mixture model.**

| Fixed effect | Intercept (se) | Linear (se) | Quadratic (se) | Cubic (se) |
| --- | --- | --- | --- | --- |
| class1 | 0(NA) | -1.04265 (0.04184) | 0.07605(0.00337) | -0.00139(0.00007) |
| class2 | -3.34451(0.16663) | 0.02022(0.01762) | 0.00018(0.00162) | -0.00001(0.00004) |
| class3 | -0.14156(0.22143) | -2.10069(0.05787) | 0.30808(0.00938) | -0.01029(0.00040) |
| Participating province | 0.08586(0.08558) | -0.06131(0.01959) | 0.00566(0.00178) | -0.00010(0.00004) |
| Random effects: variance-covariance matrix |  |  |  |  |
| $\sigma_{ⅈnt}^{2}$ | 2.017 |  |  |  |
| $\sigma_{linear slope}^{2}$ | 0.009 |  |  |  |
| $\sigma_{quadratic slope}^{2}$ | < 0.001 |  |  |  |
| $\sigma_{cubic slope}^{2}$ | < 0.001 |  |  |  |
| $\sigma_{error}^{2}$ | 1.000 |  |  |  |
| Parameters of the link function |  |  |  |  |
| Linear 1 (intercept) | 2.26345(0.07743) |  |  |  |
| Linear 2 (std err) | 0.43062(0.00282) |  |  |  |

Note: se = standard error
