# Supplementary material for: Trajectories of perioperative serum carcinoembryonic antigen and colorectal cancer outcome: A retrospective, multicenter longitudinal cohort study
Source: Clin Transl Med. 2021 Jan 21;11(2):e293. doi: 10.1002/ctm2.293 (PMC7818970; doi:10.1002/ctm2.293)
Supplement: Supplementary file 15 — SUPPORTING INFORMATION [file CTM2-11-e293-s015.docx]

**Supplementary Information.**

**Patients methods**

The ethics committee approved this multicenter retrospective study of each participating hospital. The requirement for informed consent was waived by the board, owing to the study's retrospective nature. All the patient data in the survey were anonymized.

**Patients**

All consecutive CRC patients without neoadjuvant treatment undergoing curative resection for stage I to III colorectal adenocarcinoma between January 2011 and February 2019 from three hospitals in China were retrospectively identified. We included participants if preoperative serum CEA data and at least three postoperative serum CEA measurements taken within three years after surgery were both available. The study flowchart, including the inclusion and exclusion criteria, is shown in Figure 1A.

**Serum CEA determination**

Preoperative serum CEA was defined as the CEA value closest to the time of surgery within four weeks before surgery, and postoperative serum CEA included the CEA value within three years after surgery. Repeat CEA tests were excluded. All CEA measurements were made by use of a chemiluminescence immunoassay using the COBAS 800 e602 immunoassay analyzer (Roche Diagnostics, Tokyo, Japan) at Yunnan Cancer Hospital, Alinity i immunoassay analyzer (Abbott Diagnostics, Chicago, USA) at the Sixth Affiliated Hospital of Sun Yat-sen University, and UniCel DxI 800 immunoassay analyzer (Beckman Coulter, USA) at Guangdong Provincial People's Hospital, following World Health Organization standard methods (code 73/601).^1^

**Surveillance protocol and outcome**

The surveillance protocol was detailed in our previous study.^2^ In this study, the follow-up ended on June 30, 2020. The primary endpoint was overall survival (OS). The secondary endpoint was recurrence-free survival (RFS), and recurrence included the local recurrence and distant metastases. The RFS time was defined as the time from surgery to a confirmed recurrence. All recurrent cases were confirmed via histology of a biopsy sample or positive imaging.

**Covariates**

Covariates included age, sex, preoperative CEA, surgical approach (open resection or laparoscopic resection), primary site, tumor differentiation, TNM stage, lymph node yield, mucinous (colloid) type, lymphovascular invasion, perineural invasion, and adjuvant chemotherapy.

**Statistical analysis**

Because CEA levels vary significantly among individuals, we set the CEA measurement value higher than 50ng/ml (10 times the upper limit of the normal value of CEA) to 50ng/ml before performing trajectory analysis. We did this for the following three reasons: (1) Among the CEA measurement values included in our analysis, the CEA measurement value exceeding 50ng/ml accounts for 2.37% in this cohort; (2) The CEA measurement value exceeding 50ng/ml ranges from 50.03 ng/ml to 30151.00 ng/ml, which covers most of the CEA variation; (3) In the cox analysis of preoperative CEA with outcomes, we found that its HR associated with OS and RFS remains unchanged when the preoperative CEA exceeds 50 mg/ml (Figure S2).

We used a latent class growth mixed model (LCGMM) to determine perioperative CEA trajectories,3 which assumed that the population was heterogeneous and grouped the population's trajectories by estimating the discrete potential categories. To ensure enough people to fit the trajectory at each time point, we only described the CEA trajectories within three years after surgery. And to ensure the correctness of grouping and the accuracy of parameter estimation, only patients with preoperative serum CEA data and at least three postoperative serum CEA measurements were included in the trajectory analysis.

The R package lcmm (version 1.9.2) was used to perform LCGMM, setting the log-transformed CEA as a function of time (months between each CEA measurement date and the surgery date). Considering the pattern difference between regions, we also included the participating province and its interaction with time polynomials in our model. When the LCGMM model was fitted, we assessed the polynomial function of linear, quadratic and cubic, and tried the grouping number from 1 to 5 in each function form. To avoid convergence towards local maxima, LCGMM models with 2 to 5 classes were performed several times with different sets of random starting values based on the 1-class model. The model fitting process and the comparison of trajectories with 2 to 5 groups were shown in Table S9. An optimal fit model was selected according to the following criteria: ^4^(1) significant improvement of the model in Bayesian information criterion; (2) high mean posterior probabilities (>0.7); (3) no less than 5% participants in any single trajectory class.

Finally, the best fitting model based on the above criteria was cubic trajectories of 3 groups, and the final model was described as:

$${CEA}_{ij}|_{c_{i-g}}=\left( v_{0g}+v_{0g*p}+u_{0ig} \right)+\left[ v_{1g}+v_{1g*p}*{province}_{i}+u_{1ig} \right]time+\left[ v_{2g}+v_{2g*p}*{province}_{i}+u_{2ig} \right]{time}^{2}+\left[ v_{3g}+v_{3g*p}*{province}_{i}+u_{3ig} \right]{time}^{3}+\varepsilon_{ij}$$

Where $v=\left( v_{0g},v_{1g},v_{2g},v_{3g},v_{0g*p},v_{1g*p},v_{2g*p},v_{3g*p} \right)$ is a vector of fixed-effect parameters in the group $"g"$, $u={(u}_{0ig},u_{1ig},u_{2ig},u_{3ig})$ is a vector of random effect parameters of the individual $"i"$ in the group $"g"$, $\varepsilon_{ij}$ is an unknown error term. The curve parameters of fixed and random effects in the three trajectory groups were shown in Table S10.

Characteristics across different trajectory groups were compared using ANOVA or Kruskal–Wallis tests for continuous variables and χ^2^ statistics for categorical variables.

We first estimated the OS and RFS for each trajectory group using the Kaplan-Meier method. The differences in OS and RFS among the three groups were compared using the log-rank test, respectively. We evaluated the trajectory group membership associations with the outcome and calculated the hazard ratios (HR) and 95% confidence interval (CI) by the Cox regression model. Three models were used: model 1 being unadjusted; model 2 adjusting model 1 for age, sex, and preoperative CEA; model 3 adjusting model 2 for the primary site, surgical approach, lymph node yield, pathology stage, tumor differentiation, histological type, mucinous (colloid) type, lymphovascular invasion, perineural invasion, and adjuvant chemotherapy.

To test the risk estimates' robustness, we used two additional sensitivity analyses (fitting the above three models). A frailty model analysis was performed for OS and RFS by introducing random effects in the model to account for associations and unobserved heterogeneity due to different centers' participation.^5^ A competing risk analysis was done for RFS because of possible confounding from deaths as a result of other causes.^6^

To search for potential heterogeneity sources, subgroup analyses were performed by participating province, sex, primary site, surgical approach, cancer stage, tumor differentiation, lymph node yield, and adjuvant chemotherapy, with tests for interaction by the Cox regression model.

The relative importance of each parameter to survival risk was assessed using the χ2 from Harrell’s rms R package.

The R software (version 3.6.3; [http://www.R-project.org)](http://www.R-project.org)a) was used to perform all statistical analyses. The reported statistical significance levels were all two-sided, with statistical significance set at a *P*-value <0.05. Mean values ± standard deviations (SD) (normal distribution) or median (quartile) (skewed distribution) were provided for continuous variables, whereas frequencies and percentages were provided for categorical variables.

**Reference：**

1. Laurence DJ, Turberville C, Anderson SG, Neville AM. First British standard for carcinoembryonic antigen (CEA). *Br J Cancer.* 1975;32(3):295-299.

2. Li Z, Li S, Liang Y, et al. Predictive Value of Postoperative Peripheral CD4+ T Cells Percentage in Stage I-III Colorectal Cancer: A Retrospective Multicenter Cohort Study of 1028 Subjects. *Cancer Manag Res.* 2020;12:5505-5513.

3. Proust-Lima C, Philipps V, Liquet B. Estimation of extended mixed models using latent classes and latent processes: the R package lcmm. *J Stat Softw.* 2015;78(2):1-56.

4. Andruff H, Carraro N, Thompson A, Gaudreau P, Louvet B. Latent Class Growth Modelling: A Tutorial. *Tutor Quant Methods Psychol.* 2009;5(1):11-24.

5. Ha ID, Sylvester R, Legrand C, MacKenzie G. Frailty modelling for survival data from multi-centre clinical trials. *Stat Med.* 2011;30(17):2144-2159.

6. Fine JP, Gray RJ. A Proportional Hazards Model for the Subdistribution of a Competing Risk. *J Am Stat Assoc.* 1999;94(446):496-509.
